# When Marriage Hurts: A Literature Review of Embedded Jewellery Ring Injuries

**DOI:** 10.7759/cureus.11624

**Published:** 2020-11-22

**Authors:** Sameem Tak, Esmee Irvine, Muzamil Baba, Vinayak Ghanate, Hamidreza Khairandish

**Affiliations:** 1 Trauma and Orthopaedics, University Hospitals of Leicester, Leicester, GBR; 2 Plastic Surgery, Leeds General Infirmary, Leeds, GBR; 3 Trauma and Orthopaedics, Kettering General Hospital, Kettering, GBR

**Keywords:** hand injury, ring injury, embedded ring, trauma, tourniquet

## Abstract

A ring is traditionally worn as a symbol of love and affection or as decorative ornamental jewellery. However, rings are not without risk. The spectrum of danger can range from debilitating avulsion injuries to simple contact dermatitis. Unknown to many, an unusual rarity exists; previous authors have termed this entity ‘embedded ring syndrome’. We sought to review the literature and collate evidence on the common features of this syndrome.

A literature review was performed on cases reported from 1947 to 2017 accessed through the healthcare database advanced search (HDAS). A total of 28 cases were analysed for demographics, symptomatology and operative techniques. Overall, 64.3% were females, and 50% had a psychiatric comorbidity. There was a causative event preceding the injury in 35.7% of cases; 71.4% had a reduced range of movement or reported a stiff finger and 32.1% had reduced sensation. The majority of patients underwent ring removal and primary closure, without documentation as to whether neurovascular bundles and tendons were visualised. Embedded ring injuries are rare. Consequently, information is sparsely available regarding its natural history and management. The hand surgeon’s approach requires an understanding that the chronicity of these injuries can have a significant traumatic impact on the structures of the finger.

## Introduction and background

It is believed that ancient Egyptians first established the custom of ring bearing to reflect the eternity of their marriage: impervious and unbroken. Legend has it that they bore rings on the fourth finger as *vena amoris* connected this digit directly to the heart. Such a vein does not exist; indeed, the circulatory system was unknown at the time [1,2].

**Figure 1:**
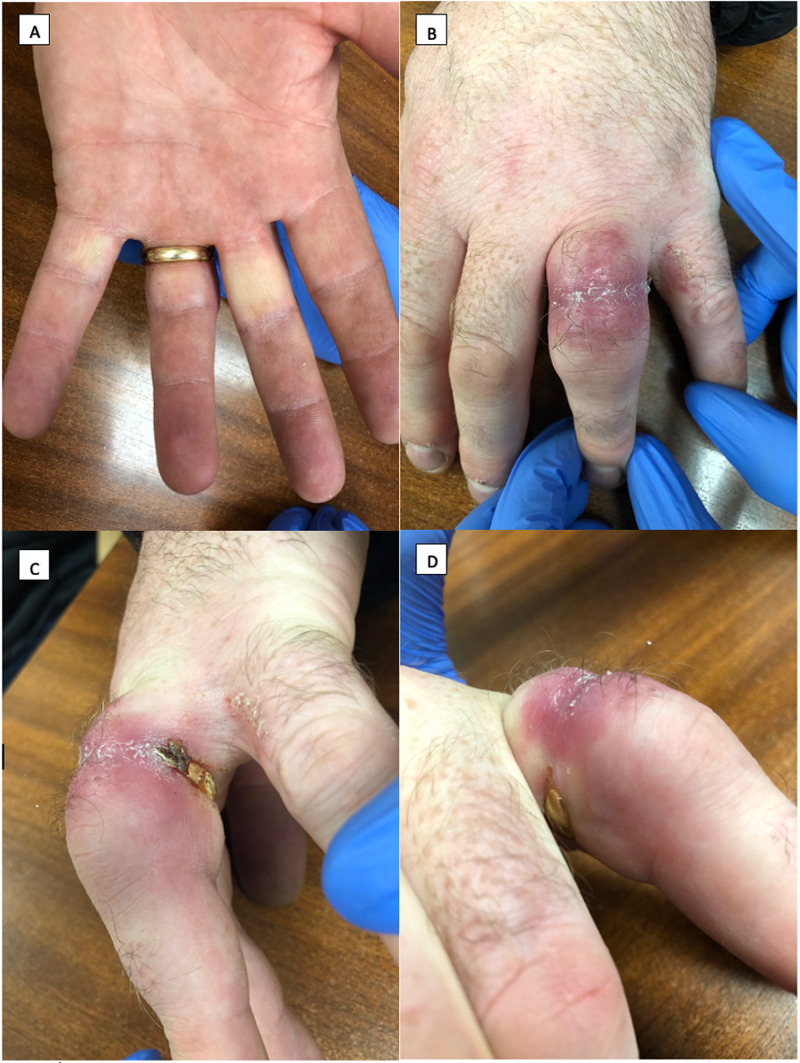
(A-D) Example of an embedded ring injury

Today, many people choose to wear a ring or ‘wedding band’, which is usually forged from metal. Commonly, ring entrapment can occur secondary to the swelling of a digit. It is unmoveable past the proximal interphalangeal joint secondary to pregnancy, allergic reaction or infection, or simply as a result of a tightfitting ring. There are well-established methods for entrapped ring removal in the emergency department such as the winding technique, which uses thread to compress the finger, or using manual ring cutters to saw the ring [3]. Rarely, they can result in complex traumatic avulsion injuries to the hand when the ring is caught on an object and forcefully pulled. These injuries can necessitate a range of treatments, from simple wound closure through to microvascular repair and amputation [4]. Rarer still, a ring can become embedded into the soft tissue of a digit to the extent that it is not at all visible to the eye. This unusual phenomenon has previously been described as ‘embedded ring syndrome’ [5]. An embedded ring can be seen as the re-epithelialisation of skin over any part of the ring resulting in the formation of a skin bridge. Figure 1 shows an example of an embedded ring. The literature is limited to sporadic case reports, and the majority of authors have presented their cases in the context of extreme rarity. Within this review, we aim to amalgamate the available data and identify the common features.

## Review

We utilised the National Institute for Health and Care Excellence (NICE) Healthcare Database Advanced Search (HDAS) via OpenAthens to search PubMed, MEDLINE, EMBASE and EMCARE databases from their inception until August 2020. The following search terms were used: ‘embedded ring injury/injuries’ or ‘ring injury/injuries’ or ‘embedded ring syndrome’ both independently and combined with ‘ulceration’ ‘erosion’ ‘digit’ ‘finger’ or ‘retained.’ Articles were included for patients of any age range and demographic. Excluded articles were those that described ring injuries without any epithelialisation/’skin bridge’ over the ring, articles relating to ring entrapment rather than embedded rings and articles not written in English text.

**Figure 2:**
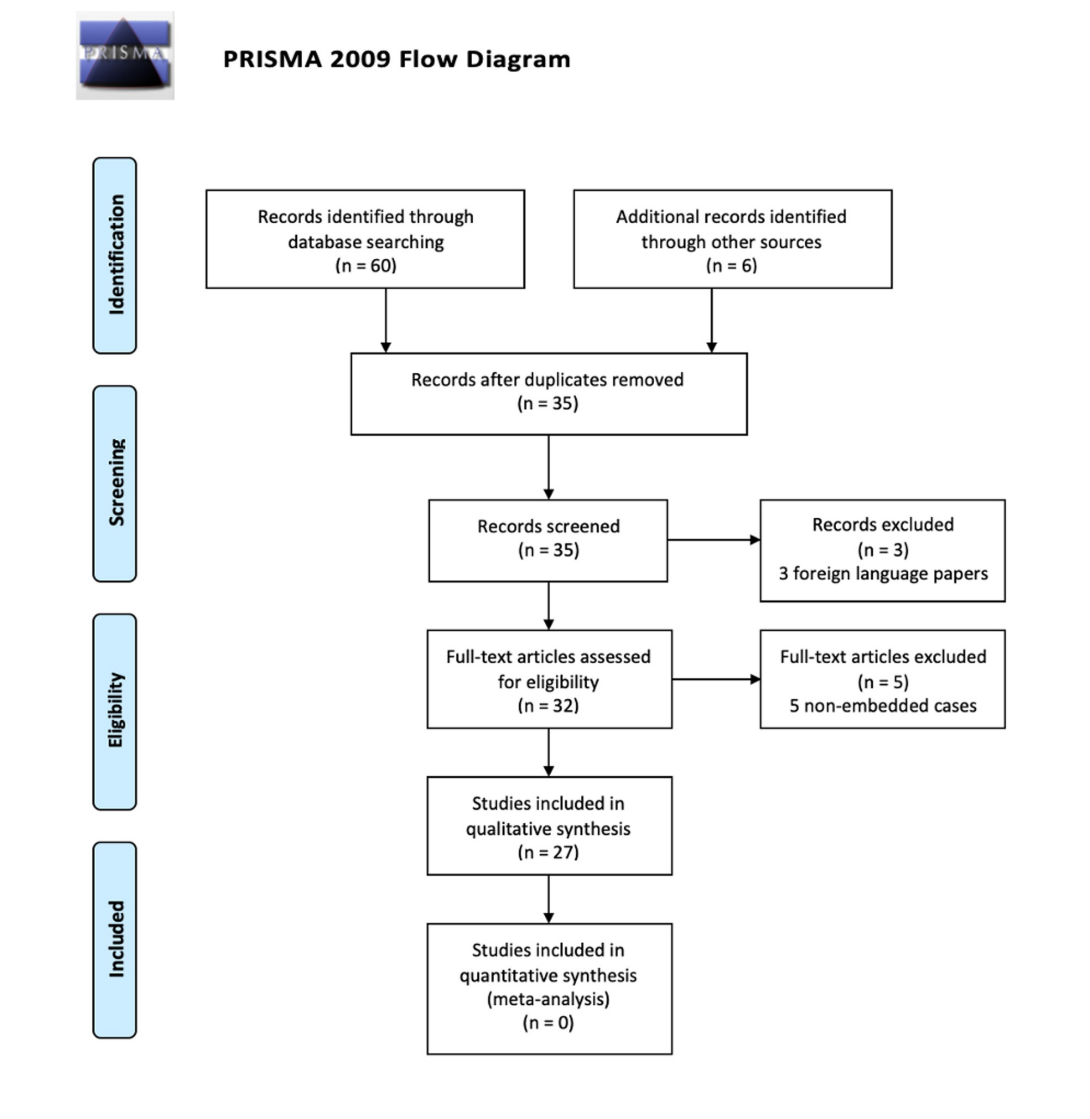
Outline of article selection process Flow diagram of the Preferred Reporting Items for Systematic Reviews and Meta-Analyses (PRISMA) process.

Figure 2 shows an outline of the systematic literature search that was carried out in accordance with the Preferred Reporting Items for Systematic Reviews and Meta-Analyses System (PRISMA) statement for study selection [6]. Two authors independently screened the 60 articles that were retrieved through the database search. The references of retrieved articles were traced for citations missed by the electronic search; this yielded a further six articles. Thirty one irrelevant and duplicate articles were removed. Three articles were excluded as they were presented in a foreign language. After screening, 32 full-text articles were selected for further evaluation: five articles were excluded as they were non-embedded cases. A total of 27 were finally put forward for analysis; 26 articles presented one case each and one article presented two cases. There was no disagreement in study selection between authors.

A total of 27 reports were analysed dating from 1947 to 2017 [5,7-32]. Given its unusual and rare nature, some of the information reported was sporadic and without structure. Information regarding patient demographics, symptomatology and operative techniques were collected where available. Table 1 shows the patient demographics and any reported causative event, as well as the reported length of time over which the injury had occurred.

**Table 1:** Patient demographics Note: Unrecorded data are represented by '-'

Author	Gender	Age	Medical Comorbidity	Psychiatric Comorbidity	Causative Event	Duration of Symptoms	No. of Digits Involved	Digit(s)
Awan et al. [7]	F	16	-	Intellectual disability	No	-	1	Index finger
Balakrishnan and Nyitray [8]	F	24	-	Intellectual disability	-	-	1	Ring finger
Bennett et al. [9]	M	13	-	-	No	1 month	1	Ring finger
Zeng et al. [10]	M	18	No	yes; secondary to amphetamine abuse	Trauma	2 weeks	1	Middle finger
Deshmukh and Stothard [11]	M	22	-	Yes; unspecified	-	-	1	Middle finger
Drake et al. [12]	F	39	-	Yes; unspecified	-	-	1	Middle finger
Drewniany et al. [13]	F	62	Diabetes mellitus, cerebrovascular accident	-	No	-	1	Ring finger
Fraser and Jamison [5]	M	28	-	-	No	-	1	Ring finger
Freedman [14]	F	73	Heart failure, diabetes mellitus, anaemia	-	Trauma	9 years	1	Ring finger
Hove and Odland [15]	F	36	-	Intellectual disability	-	31 years	1	Ring finger
Kattan et al. [16]	F	17	-	-	Trauma	3 months	1	-
Kumar et al. [17]	M	49	-	Schizophrenia, depression	Trauma	3 months	1	Index finger
Kuschner et al. [18]	M	44	-	Yes; unspecified	-	-	2	Ring and middle finger
Kuschner et al. [18]	M	48	-	Schizophrenia	-	-	1	Thumb
Langridge et al. [19]	F	45	No	No	Insect Bite	Several months	1	Ring finger
Leung and Ip [20]	M	71	-	No	-	-	1	Ring finger
Magos & Sheikh [21]	F	71	Subarachnoid haemorrhage	No	Trauma	9 weeks	1	Ring finger
Moore et al. [22]	M	41	HIV	Schizophrenia	-	Several years	1	Index finger
Prasad et al. [23]	F	7	-	-	-	4 years	1	Index finger
Reguesse et al. [24]	F	69	No	No	No	-	1	Ring finger
Rohilla et al. [25]	M	22	-	No	Trauma	1 week	1	Middle finger
Saltz et al. [26]	F	23	-	Yes; unspecified	-	Several months	1	Little finger
Shafiroff [27]	F	29	-	Intellectual disability	Rapid weight gain	4 months	1	Ring finger
Sleilati et al. [28]	F	63	-	Intellectual disability	-	-	1	Ring finger
Uemura et al. [29]	F	73	No	Yes; unspecified	Trauma and rapid weight gain	10 years	1	Ring finger
Unlü et al. [30]	F	54	No	No	No	1 year	3	Ring, middle and index finger
Witt [31]	F	8	No	No	Trauma	3 months	1	Ring finger
Woodhouse [32]	F	47	-	-	-	3 months	1	Ring finger

Patients ranged from 7 to 73 years of age. Excluding the three paediatric patients aged 7, 8 and 13, there was an average adult age of 43.3 years; 64.3% were female (n=18) and 35.7% were male (n=10). Also, 64.3% of authors (n=18) did not comment on any medical comorbidities, 21.4% reported none (n=6) and 14.3% stated patient comorbidities (n=4).

Of the 28 patients, 50% (n=14) had a psychiatric comorbidity, whilst 25% (n=7) had none; the remaining 25% (n=7) of reports did not make any reference to psychiatric comorbidity. The diagnoses included intellectual disability, schizophrenia, mental illness secondary to drug abuse and depression. Five authors referred to a psychiatric comorbidity but did not specify the diagnosis. None of the three paediatric cases reported a psychiatric diagnosis.

Of the patients, 35.7% (n=10) reported a causative event correlating to the onset of symptoms. This included a clear history of traumatic injury to the finger, rapid weight gain and insect bite. Also, 21.4% (n=6) reported no obvious preceding event, and for the remaining 42.9% (n=12), no information was given or a history could not be obtained due to psychiatric comorbidity.

Overall, 60.7% (n=17) reported a time duration of symptoms before presentation whilst 39.3% (n=11) did not. Three authors did not objectively quantify the reported durations, instead they referred to the time period as ‘several months/years’. Of the patients, 7.1% (n=2) had embedded ring symptoms develop over less than one month, 32.1% (n=9) developed over one month to one year and 21.4% (n=6) developed over the course of more than one year; 39.3% (n=11) did not report a duration of symptoms.

**Figure 3:**
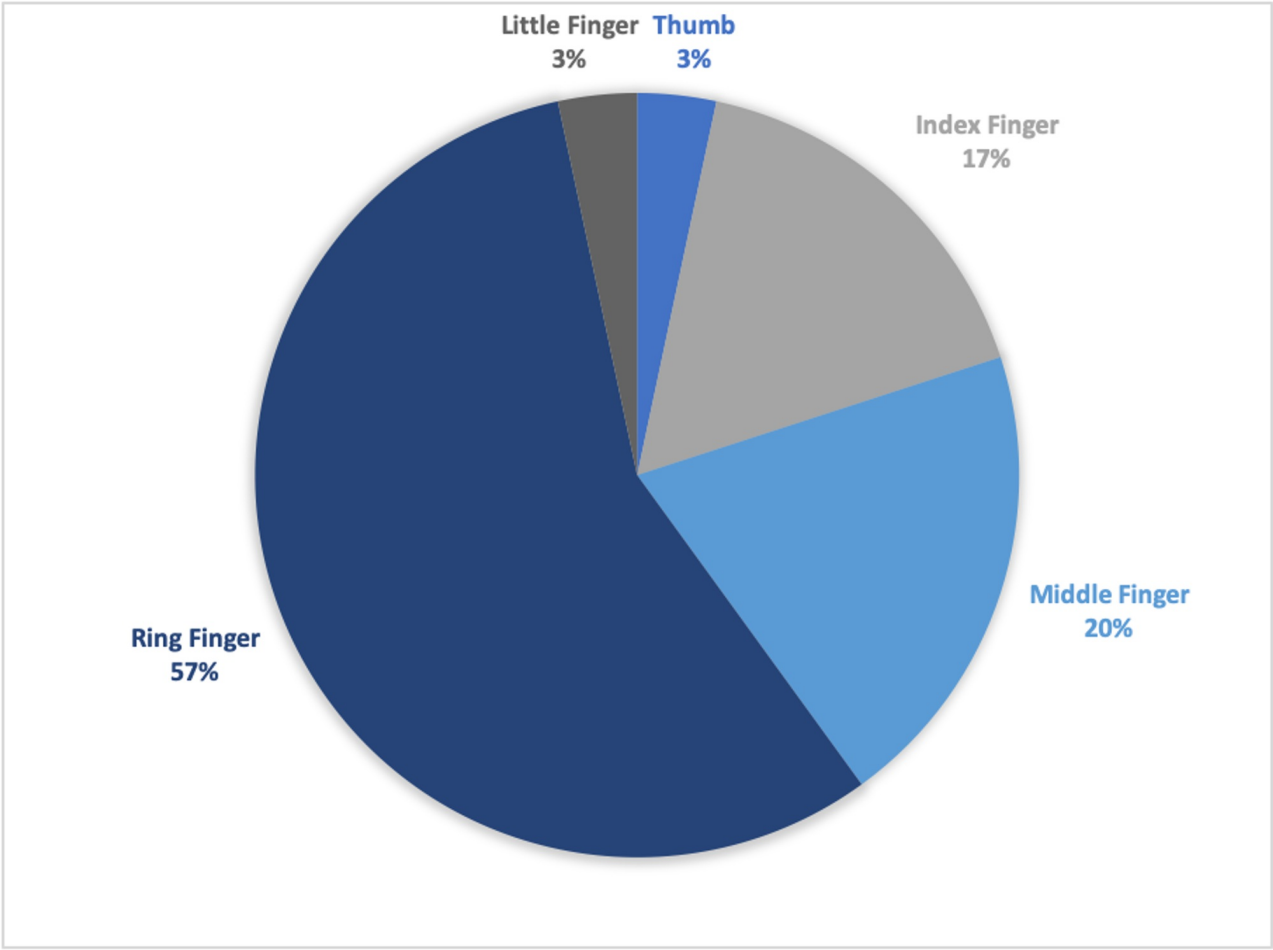
Pie chart showing embedded ring injuries by the digit involved

There were two cases of rings embedded on multiple fingers, and the remainder of cases involved only one digit. The ring finger was the most common digit to have an embedded ring. Figure 3 shows the distribution of embedded rings by finger involved.

Table 2 shows the clinical findings recorded of the patients with embedded rings. The commonest position of a skin bridge was on the volar aspect of the finger (67.9%, n=19). Dorsal skin bridges occurred in 10.7% (n=3). Completely circumferential skin bridges (i.e. an invisible ring) occurred in 10.7% (n=3). Position of skin bridge was not clear in two cases (7.1%). One case (3.6%) had near-complete embedding: skin bridging on the dorsal, volar and ulnar border of the finger but visibility of the ring on the radial border. Moreover, 35.7% (n=10) had pain/discomfort, 21.4% (n=6) had no pain/discomfort and 42.9% (n=12) did not report upon this finding.

**Table 2:** Comparison of clinical findings between reported cases IPJ, interphalangeal joint; CRT, capillary refill time Note: Unrecorded data are represented by '-'

Author	Position of Skin Bridge on Finger	Discomfort/Pain in Finger	Vascular Status	Distal Sensation	Bony Involvement	IPJ Movement	Erythema	Swelling	Signs of Infection
Awan et al. [7]	Volar, dorsal and ulnar border	No	Normal	Normal	No	Normal	Yes	Yes	No
Balakrishnan and Nyitray [8]	-	-	Normal	Normal	No	Normal	-	-	Yes
Bennett et al. [9]	Complete circumferential	-	Normal	Normal	Yes	Reduced	-	Yes	Yes
Zeng et al. [10]	Volar	Yes	Normal	Reduced	-	Reduced	Yes	Yes	Yes
Deshmukh and Stothard [11]	Volar	Yes	Normal	Normal	No	Reduced	-	Yes	No
Drake et al. [12]	Volar	-	Normal	Normal	Yes	Reduced	-	Yes	No
Drewniany et al. [13]	Volar	-	Normal	Reduced	Yes	Reduced	-	-	-
Fraser and Jamison [5]	Volar	Yes	Normal	Normal	No	Reduced	-	Yes	-
Freedman [14]	Volar	-	-	-	Yes	Reduced	-	Yes	No
Hove and Odland [15]	Dorsal	Yes	Normal	Normal	Yes	Reduced	-	Yes	Yes
Kattan et al. [16]	Volar	-	-	-	Yes	-	-	-	-
Kumar et al. [17]	Volar	Yes	Delayed CRT of 5 seconds	Reduced	-	Reduced	Yes	Yes	-
Kuschner et al. [18]	Dorsal	-	Normal	Reduced	No	Reduced	-	Yes	No
Kuschner et al. [18]	Volar	-	Normal	Normal	-	Reduced	-	-	-
Langridge et al. [19]	Volar	No	Normal	Normal	No	Normal	No	Yes	No
Leung and Ip [20]	Volar	Yes	Normal	Normal	Yes	Reduced	-	Yes	Yes
Magos and Sheikh [21]	Volar	No	Normal	Normal	-	Normal	-	-	No
Moore et al. [22]	-	-	Normal	Reduced	No	Reduced	-	Yes	No
Prasad et al. [23]	Dorsal	No	Normal	-	Yes	Reduced	-	Yes	-
Reguesse et al. [24]	Volar	Yes; minimal	Normal	Reduced	No	Reduced; stiff	Yes	Yes	No
Rohilla et al. [25]	Complete circumferential	Yes	Normal	Normal	No	-	-	-	-
Saltz et al. [26]	Volar	No	Normal	Normal	-	-	No	Yes	Yes
Shafiroff [27]	Volar	-	Delayed CRT	Normal	-	-	Yes	Yes	Yes
Sleilati et al. [28]	Volar	Yes	Normal	Hyperaesthesia	Yes	Reduced	Yes	Yes	-
Uemura et al. [29]	Volar	-	Normal	Reduced	No	Reduced; stiff	Yes	Yes	Yes
Unlü et al. [30]	Volar	-	Normal	Reduced	Yes	Reduced; stiff	Yes	Yes	No
Witt [31]	Complete circumferential	No	Normal	Reduced	Yes	Reduced	Yes	Yes	No
Woodhouse [32]	Volar	Yes	Normal	Normal	-	Reduced	Yes	Yes	Yes

The vast majority had normal vascular supply in the digit (85.7%; n=24), two reported delayed capillary refill times (7.1%) and 7.1% (n=2) did not report upon vascular status. Distal to the site of injury, 53.6% (n=15) had normal sensation and 32.1% (n=9) had reduced sensation. And, 3.6% (n=1) had hyperaesthesia, 10.7% (n=3) did not report upon sensation, 39.3% (n=11) had a ring embedded into bone in addition to soft tissues, 35.7% (n=10) did not involve bone and 25% (n=7) were unreported.

Of the patients, 71.4% (n=20) had a reduced range of movement or reported a stiff finger. Normal range of movement (ROM) was described in 14.3% (n=4). Erythema was present in 35.7% (n=10) and absent in 7.2% (n=2). All cases that commented upon swelling stated that it was present in the offending finger (78.6%; n=22); 32.1% showed signs of infection (n=9) and 39.3% showed no signs of infection (n=11).

Table 3 shows the intraoperative findings. The available data on this aspect was sparse. A majority of authors reported using ring cutters to release the ring, with two using wire cutters and one using a tapered fissure burr. Only seven authors stated that they explored the wounds. Five authors commented on tendon integrity, with three reporting some degree of tendon rupture. Two authors reported collateral vessel formation as a result of the embedded ring, with Awan et al. showing a neurovascular bundle traversing over the top of an embedded ring [7].

**Table 3:** Reported intraoperative data NVB, neurovascular bundle(s); ROM, range of movement; FDP, flexor digitorum profundus; FDS, flexor digitorum superficialis Note: Unrecorded data are represented by '-'

Author	Mode of Anaesthetic	Incision	Wound Exploration	Instrument for Ring Removal	Intraoperative Course	Follow-Up
Awan et al. [7]	Regional	Yes	Yes	Ring cutters	Intact NVB and flexor tendons, neovascularisation, growth of NVB over top of ring: repair not required	Residual stiffness
Balakrishnan and Nyitray [8]	-	-	-	-	-	Return of normal ROM
Bennett et al. [9]	-	Yes	Yes	Ring cutters	Intact NVB	Return to near-normal ROM
Zeng et al. [10]	-	No	No	Ring cutters	-	-
Deshmukh and Stothard [11]	-	-	-	Ring cutters	-	-
Drake et al. [12]	General	Yes	No	Ring cutters	-	Restricted ROM
Drewniany et al. [13]	-	-	-	Ring cutters	-	Restricted ROM, residual sensory disturbance
Fraser and Jamison [5]	-	-	-	Ring cutters	Intact NVB, intact flexor and extensor tendons	-
Freedman [14]	-	-	-	-	-	Restricted ROM
Hove and Odland [15]	Local	No	No	Ring cutters	-	Restricted ROM
Kattan et al. [16]	-	-	-	-	-	-
Kumar et al. [17]	-	Yes	No	Ring cutters	-	Return of normal sensation, restricted ROM
Kuschner et al. [18]	-	-	-	-	-	-
Kuschner et al. [18]	General	Yes	-	Ring cutters	-	Restricted ROM
Langridge et al. [19]	General	Yes	Yes	Wire cutters	-	-
Leung and Ip [20]	Local	Yes	Yes	Ring cutters	Ruptured FDS, ruptured extensor digitorum, normal radial NVB (ulnar NVB not explored): tenolysis and tenosynovectomy of flexors and extensors	Return of normal ROM
Magos and Sheikh [21]	Local	No	No	Ring cutters	-	Normal
Moore et al. [22]	General	No	No	Tapered fissure burr	-	Improved ROM, residual sensory disturbance
Prasad et al. [23]	General	-	-	-	-	Improved ROM
Reguesse et al. [24]	-	No	No	Ring cutters	-	Residual stiffness, residual sensory disturbance
Rohilla et al. [25]	Local	-	-	Ring cutters	-	Normal
Saltz et al. [26]	Local	Yes	No	Wire cutters	-	-
Shafiroff [27]	General	No	No	Ring cutters	-	-
Sleilati et al. [28]	General	-	-	-	-	Return of normal sensation, restricted ROM
Uemura et al. [29]	Regional	Yes	Yes	None required	Ruptured FDP, compressed NVB: patient refused repair	Return of normal sensation, restricted ROM
Unlü et al. [30]	-	-	-	-	Neovascularisation	Residual stiffness
Witt [31]	-	Yes	Yes	Ring cutters	-	Residual stiffness
Woodhouse [32]	General	Yes	Yes	-	Partially ruptured FDS: no repair	Return of normal ROM

Twenty authors reported follow-up findings at varying lengths of time postoperatively. Six patients had an improvement in their interphalangeal joint ROM/stiffness, whilst 12 showed no improvement at follow-up. Three patients had an improvement in their sensibility, whilst three had residual sensory disturbance.

The term ‘embedded ring syndrome’ has previously been used to describe the association of this injury with psychiatric illness [5]. Initial case reports in the literature pointed towards a prerequisite of mental illness to develop an embedded ring; however, as shown in our review, it is not an absolute requirement; 25% of patients were confirmed to have no mental illness.

All three of the paediatric cases had radiographic evidence of the ring eroding into the proximal phalanx [9,23,31]. A possible explanation for this unusual phenomenon may be that as the ring embeds, the child’s finger continues to grow, and the embedded ring is encompassed into the growing bone. These cases also highlight that there may be delays in presentation and diagnosis, owing to the child’s lack of insight or sense of deep embarrassment [9].

Adult cases with evidence of bony erosion reported a long duration of symptoms (9 years, 10 years and 31 years), supporting the suggestion that chronicity increases the risk of bony involvement [14-15,29]. A 17-year-old patient was found to have an embedded ring in her proximal phalanx, with a reported symptom duration of three months; this time period may have been shortened since the patient had not reached skeletal maturity [16]. We recommend that radiographs are indicated when presented with an embedded ring injury, firstly, to identify any bony involvement and, secondly, to detect any hidden rings that may not be identified on clinical examination [9,28].

There was a clear history of a causative event in 35.7% (n=10) of patients, and the most common history was one of a traumatic insult adjacent to the ring. It could logically follow that traumatic disruption of the epithelium combined with the chronic circumferential constriction of a ring provides an opening through which the ring can ulcerate down the subcutaneous layer to the bone. Reepithelialisation then occurs atop the ring. Almost all cases reported digital swelling. This is secondary to the mechanical obstruction of the ring causing venous congestion and disruption to lymphatic drainage [17]. Given the chronicity of symptoms and confounding psychiatric factors, it is likely that small traumatic events to the finger are underreported by the patient.

Two authors reported delayed capillary refill times, but there was no evidence of ischaemic necrosis in any patient, and none required amputation. The embedded ring is a rigid metal structure and is not collapsible; indeed, patients present with rings that have been embedded for many years but show no sign of vascular compromise. This is in contrast to a collapsible structure that acts as a tourniquet, such as in hair tourniquet syndrome [33]. However, the sample size of 28 patients is too small to draw a definitive conclusion that embedded rings do not lead to ischaemia. Additionally, given the thicker periosteum and open physis, the risk of ischaemia cannot be dismissed in a paediatric patient as growth occurs, which may result in the occlusion of neurovascular structures.

The majority of patients underwent surgical ring removal and primary closure, without documentation as to whether neurovascular bundles and tendons were visualised. Given that a majority of patients (71.4%, n=20) presented with reduced range of movement (which persisted at follow-up), it is possible that tendon damage was present but not visualised.

Only one author performed operative repair; tenolysis and tenosynovectomy were performed after finding ruptured flexor and extensor tendons [20]. Regarding the neurovascular bundle, compression of the bundle was the only adverse finding reported on this structure [29]. The sensory disturbance that results from the embedded ring is likely due to neuropraxia secondary to oedema and direct pressure on the nerve from the adjacent ring.

Learning points

1. When there is any traumatic injury adjacent to a ring, the ring should be removed until the injury has healed.

2. Ischaemia is unlikely to be a feature of embedded ring injuries; however, available data are sparse and a theoretical risk still exists. 

3. Embedded rings are not exclusive to the psychiatric population.

4. Radiographs should always be obtained in embedded ring injuries to identify bony involvement and hidden rings not visible on clinical examination.

## Conclusions

Embedded ring injuries are rare. Consequently, information is sparsely available regarding its natural history and management. The hand surgeon’s approach requires an understanding that the chronicity of these injuries can have a significant traumatic impact on the structures of the finger.

The responsible healthcare professional should consider the patient’s mental health status when determining whether wound exploration and structural repair should be performed. A discussion should be had with the patient regarding the possibility of tendon or nerve repair in addition to ring removal to identify the patient’s expectations. Given the preponderance of psychiatric diagnoses in the embedded ring injury population, clear treatment goals should be identified.
